# Understanding the Dynamic Aggregation in Single‐Atom Catalysis

**DOI:** 10.1002/advs.202308046

**Published:** 2024-01-29

**Authors:** Laihao Liu, Tiankai Chen, Zhongxin Chen

**Affiliations:** ^1^ School of Science and Engineering The Chinese University of Hong Kong Shenzhen Guangdong 518172 China

**Keywords:** catalyst stability, operando spectroscopies, reaction mechanism, reversible aggregation, single‐atom catalyst, structural dynamism

## Abstract

The dynamic response of single‐atom catalysts to a reactive environment is an increasingly significant topic for understanding the reaction mechanism at the molecular level. In particular, single atoms may experience dynamic aggregation into clusters or nanoparticles driven by thermodynamic or kinetic factors. Herein, the inherent mechanistic nuances that determine the dynamic profile during the reaction will be uncovered, including the intrinsic stability and site‐migration barrier of single atoms, external stimuli (temperature, voltage, and adsorbates), and the influence of catalyst support. Such dynamic aggregation can be beneficial or deleterious on the catalytic performance depending on the optimal initial state. Those examples will be highlighted where in situ formed clusters, rather than single atoms, serve as catalytically active sites for improved catalytic performance. This is followed by the introduction of operando techniques to understand the structural evolution. Finally, the emerging strategies via confinement and defect‐engineering to regulate dynamic aggregation will be briefly discussed.

## Graphical abstract

In this review, the dynamic aggregation process in single‐atom catalysts is summarized, featuring the inherent mechanistic nuances and influence on the catalytic performance. Examples of the formed clusters, rather than single atoms, as the catalytically active sites will be highlighted and tracked by typical operando techniques to understand the structural evolution.

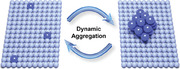

## 1 Introduction

The past decade has witnessed the explosion of research on atomically dispersed metal catalysts or “single‐atom catalysts (SACs).”^[^ ^1^, ^2^ ^]^ The interest is driven by the desire to mimic the homogeneous pathway for extremely high activity and chemoselectivity on durable, process‐friendly heterogeneous supports.^[^ ^3^ ^]^ Particularly, SACs are advantageous in their maximum metal utilization efficiency and atomically precise coordination compared to other subnanometric variants (clusters/aggregates of several atoms, nanoparticles, etc.). Extensive efforts have delivered significant progress in the areas of synthesis, characterization, applications, and mechanism insights of SACs.^[^ ^4^, ^5^ ^]^

However, downsizing to the single‐atom level brings its own set of challenges. Conventional wisdom suggests that active metal single atoms tend to agglomerate in working conditions to minimize the surface energy, which is generally due to two mechanisms at play: site‐migration and coalescence of entire nanoparticles and Ostwald ripening with the motion of atomic species from smaller to larger particles.^[^ ^6^, ^7^ ^]^ In both mechanisms, an emergence of larger particles will usually lead to the loss of catalytic activity and selectivity.^[^ ^8^ ^]^ Many strategies are proposed to prevent the aggregation of SACs by regulating the metal–substrate interaction and, more recently, to reverse the aggregation by external stimuli.^[^ ^5^ ^]^ Such reversible (dynamic) aggregation is highly desirable for the application in complex reactions that require activation of two (or more) reactants, where the single‐site mechanism of SACs is inefficient compared to the dual or multi‐site mechanism of clusters or nanoparticles.^[^ ^3^ ^]^ Understanding the dynamic structural evolution of SACs is the key to establishing the structure‐function relationship for subnanometric catalysts composed of single atoms and clusters.^[^ ^2^, ^9^ ^]^

Despite its significance, monitoring the dynamic structural evolution of SACs is not easy.^[^ ^10^ ^]^ The challenges arise from the inherent heterogeneity of supported surfaces associated with binding sites (corners, edges, and faces), defects (vacancies, terraces, and grain boundaries), and amorphous structures (hydrated layers, amorphous supports, and so on).^[^ ^11^ ^]^ Most characterization by electron microscopy is performed ex situ for the fresh and spent catalysts to achieve atomic resolution in a nanometer‐sized area.^[^ ^10^ ^]^ Spectroscopic measurements usually provide a more comprehensive view of the entire sample in the working conditions; however, averaged information about the electronic state, local coordination environment, and vibrational modes of adsorbates are collected, resulting in insensitivity in distinguishing minor species from bulk information.^[^ ^3^ ^]^ There is a trade‐off between spatial resolution at the nanoscale and time response to dynamic conditions in the state‐of‐the‐art operando techniques to uncover the real active sites during dynamic aggregation of SACs.

This review aims to provide a comprehensive understanding of the dynamic aggregation of single‐atom catalysts in working conditions. The thermodynamics and kinetics parameters collectively contribute to the intrinsic stability of SACs, while the external conditions including temperature, voltage, and reaction intermediates, induce a reversible or irreversible structural dynamism. This will consequently affect the catalytic activity, selectivity, and stability. Typical operando techniques used for monitoring dynamic evolution will also be covered, with an emphasis on identifying real active sites. Finally, we provide a complementary perspective on regulating dynamic aggregation by nano‐confinement and defect‐engineering for better catalyst designs.

## 2 The Driving Force of Dynamic Aggregation

Single metal atoms do not represent a thermodynamically stable entity on their own. The substrate supports with strong interaction to single metal atoms must be available to prevent nucleation of metal atoms to nanoparticles.^[^ ^12^ ^]^ An ideal substrate would allow a certain degree of structural flexibility for SACs to respond to dynamic catalytic cycles while retaining their integrity for stable operation.^[^ ^13^ ^]^ In the following section, we will uncover the inherent mechanistic nuances that control the dynamic profile of SACs from the thermodynamic and kinetic viewpoints.

### 2.1 Intrinsic Stability of SACs

**Figure 1:**
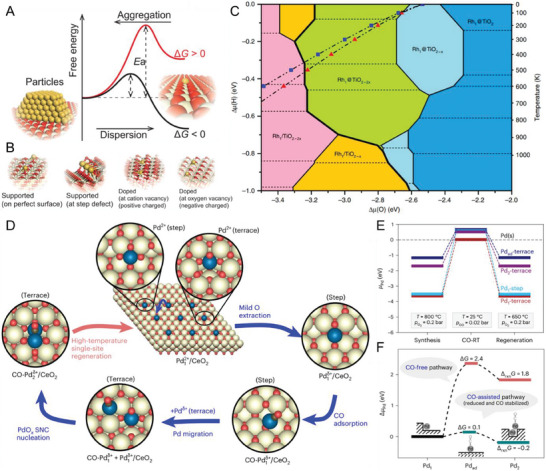
Dynamic aggregation induced by surface migration. A) Schematic illustration of free‐energy diagram of sintering and dispersion processes between Au nanoparticles and single atoms; B) Four types of single atoms on oxide support; C) Surface stability diagram for Rh single atom on TiO_2_(110) in the presence of H_2_. Axes represent the H and O chemical potentials (noted *Δµ*(H) and *Δµ*(O) in eV); D) DFT computed atomic structures of the favored Pd states on Pd‐doped CeO_2_ (111) at different operation stages; E) Evolution of the chemical potential of Pd (*µ*_Pd_) at different stages with reaction conditions relative to bulk Pd; F) Free energy landscape for Pd_1_ activation with (*p*_CO_  =  0.02 atm) and without CO at 25 °C. Reproduced with permission.^[^ ^6^, ^14^, ^22^, ^27^ ^]^ (A, B) Copyright 2018, Oxford Academic. (C‐F) Copyright 2019 and 2023, Springer Nature.

The intrinsic stability of SACs can be well explained by the thermodynamic diagram in **Figure** **1**A,B. When the free‐energy change from nanoparticles to single atoms is negative, nanoparticles can be dispersed to single atoms spontaneously, leading to the thermodynamic stability of SACs. However, even if the free‐energy change is positive, single atoms can be kinetically stable when the aggregation barrier is sufficiently high to prevent sintering.^[^ ^14^ ^]^ In this regard, the metal‐substrate interaction (or binding energy of single atoms, *E*_bind_) is a suitable descriptor to predict the thermodynamic stability of SACs. For instance, stable SACs will not be formed on the basal plane of graphene and 2H‐phase MoS_2_ owing to their weak interaction. The presence of defects (such as steps and vacancies), dopants, and surface terminations significantly improve the stability of single atoms via surface trapping.^[^ ^2^, ^15^ ^]^ Several examples of SACs with higher thermal stability than their supported nanoparticle analogs have been reported.^[^ ^16^, ^17^, ^18^, ^19^, ^20^, ^21^ ^]^

Apparently, a stronger metal‐support interaction (or *E*_bind_) implies a lower mobility of atomic species on the support, but this does not truly reflect the diffusion activation barrier (*E*_a_). This suggests that a thermodynamic metric of binding energies alone cannot explain the kinetic stability in most SAC systems. A more detailed study by Hensen et al.^[^ ^22^ ^]^ revealed the importance of the cohesive energy of bulk metal (*E*_c_) for the prediction of *E*_a_, which reflects the intrinsic chemical reactivity of single atoms. The diffusion activation barrier has a strong linear dependency on the ratio of (*E*_bind_)^2^/*E*_c_ for various SAC systems on reducible metal oxides (CeO_2_, TiO_2_), stable metal oxides (MgO, ZnO), perovskite (SrTiO_3_), and 2D materials (MoS_2_ and graphene). Indeed, the ratio of *E*_bind_/*E*_c_ serves as a correction factor to *E*_bind_ by comparing the affinity of a single metal atom to the support surface and bulk metal. This provides a simple model for screening thermodynamics to the kinetics of metal adatom on a support.^[^ ^22^ ^]^

### 2.2 Theoretical Considerations on Site Migration

From the thermodynamics aspect, the driving force of atomic migration is generally described by this equation:^[^ ^23^, ^24^ ^]^

(1) $$\Delta G_{\mathrm{mig}}=\Delta H_{\mathrm{mig}}-T\Delta S_{\mathrm{mig}}+\Delta \gamma_{\mathrm{mig}}A$$

where *ΔG*_mig_, *ΔH*_mig_, *ΔS*_mig_, and *Δγ*_mig_ are the changes in Gibbs free energy, enthalpy, entropy, and specific surface energy before and after atomic migration, respectively. *A* is the surface area, and *T* is the absolute temperature. Site migration could happen when the *ΔG*_mig_ is negative and is affected by many factors, such as the concentration‐induced energy difference, atomic bonding strength (mainly related to *ΔH*_mig_), and surface energy (related to *Δγ*_mig_) in structure features of nanocatalysts.^[^ ^23^, ^25^ ^]^

The most well‐known example is the Ostwald ripening, where large particles grow at the expense of small ones to reduce the overall surface energy.^[^ ^2^ ^]^ The driving force (related to *Δγ*_mig_) arises from the surface energy difference owing to more exposed undercoordinated atoms on the surface of small nanoparticles. The detailed process may involve the atomic dissolution from smaller particles into electrolytes and subsequent redeposition of dissolved atoms on larger particles.^[^ ^23^ ^]^ Similarly, the agglomeration process of nanocatalysts via initial detachment from support and subsequent coalescence with each other would be affected by the size as a consequence of the surface energy difference.^[^ ^2^ ^]^ Apart from particle size, chemical composition (related to *ΔH*_mig_), facet, and curvature will have a strong influence on the free energy change.

The kinetics feature of atomic migration is less studied due to the difficulty in experimental validation. In principle, it can be described by the Arrhenius equation:^[^ ^23^ ^]^

(2) $$D_{\mathrm{mig}}=D_{0}\exp\left(-E_{\mathrm{mig}}/RT\right)$$

where *D*_mig_ is the migration rate constant, *D*_0_ is the pre‐exponential factor, *E_mig_* is the atomic migration energy barrier, and *T* is the absolute temperature. As mentioned above, the activation energy barrier is closely related to the metal–substrate interaction (*E*_bind_) and the cohesive energy of bulk metal (*E*_c_), which depends on the type of element, facet, defect, and reaction intermediate. External stimuli in working conditions, including temperature, electric field, and adsorbates, facilitate the atomic migration of single atoms.^[^ ^23^, ^25^ ^]^ For example, high temperature induces a larger amplitude of atomic vibration near the equilibrium position, thereby increasing the possibility to cross the migration barrier. External electrical fields can drive the atomic diffusion or migration toward specific directions and even cause the uphill diffusion behavior.^[^ ^23^ ^]^

It should be pointed out that SACs can undergo dynamic structural transformations in both geometric (e.g., aggregation) and electronic structures in a catalytic cycle. The changes in local coordination environments (e.g., type and number of coordinated atoms) by bond dissociation and oxidation states by polarization or distortion of its electron clouds can interplay for a macroscopical behavior of structural evolution.^[^ ^3^ ^]^ Such evolution could be progressive, irreversible, and easily detected by ex situ techniques. By contrast, it may occur discreetly and transiently under reaction conditions, requiring the use of operando methods to understand the underlying mechanism.^[^ ^1^ ^]^

### 2.3 Dynamic Response in the Single‐Component Atmosphere

Subnanometric metal species can evolve after exposure to a single component atmosphere (non‐reactive atmosphere), such as O_2_, H_2_, CO, and H_2_O, driven by their thermodynamic and kinetic stability.^[^ ^26^ ^]^ A representative example would be the restructuring of Pt nanoparticles into single atoms in air at elevated temperature (800 °C) or sintering into larger particles under an inert atmosphere.^[^ ^17^, ^18^ ^]^ Such particle redispersion is triggered by the formation of PtO_2_ (the dominant surface species) in an oxidative environment owing to the much lower evaporation free energy of PtO_2_ from an oxygen pre‐covered Pt(221) step compared to that of a single Pt atom from a Pt(221) step (−0.61 eV vs 4.00 eV). Therefore, it is energetically strongly unfavorable for redispersion in an inert atmosphere due to the strong covalent metal‐support interaction of Pt_1_ to the surface atom.^[^ ^17^ ^]^ In a reductive atmosphere (such as H_2_), Pt single atoms aggregate into clusters and nanoparticles, occurring at a relatively low temperature of 200 °C on the surface of non‐reducible Al_2_O_3_. The fraction of single Pt atoms decreases and eventually vanishes when raising the reduction temperature to 300 °C and 450 °C.^[^ ^9^ ^]^ In another study, the influence of H_2_O (moisture) on the dynamic profile of the CuAl sample was carefully investigated by reoxidizing the reduced samples in dry or wet air. The H_2_ reduction and dry air re‐oxidation promote the agglomeration of small CuO clusters and single Cu^2+^ sites. In contrast, wet air reoxidation could partially recover these CuO species to their initial state, with a small number of CuO species mildly agglomerated and easily reduced.^[^ ^7^ ^]^ Therefore, pretreatment in a single‐component atmosphere profoundly influences the chemical states and local coordination environment of the catalyst.

### 2.4 Dynamic Response to Reactive Working Conditions

Given the structural dynamism of SACs, their stability should be rigorously examined in reactive working conditions (e.g., in the presence of adsorbates). In general, SACs do not have static local coordinations but can switch from inactive to active structure (or vice versa) under reaction conditions.^[^ ^26^ ^]^ For instance, the preferred local coordination of Rh single atoms was investigated on TiO_2_ during calcination in O_2_, reduction in H_2_, CO adsorption, and reverse water gas shift (RWGS) reaction conditions.^[^ ^27^ ^]^ Theoretical and experimental studies clearly demonstrated the dynamic response of Rh single atoms in the local coordination and reactivity to various redox conditions. As shown in Figure 1C, the surface stability diagram in H_2_ indicates that the preferred structure on the Rh_1_/TiO_2_ (110) surface is governed by the H and O chemical potentials. Blue, light blue, and green regions denote the preferential substitution of six‐coordinated surface Ti with zero, one, or two O vacancies by a Rh atom (Rh_1_@TiO_2_, Rh_1_@TiO_2‐_ *_x_*, Rh_1_@TiO_2‐2_ *_x_*). Meanwhile, the supported Rh structure is favored (Rh_1_/TiO_2‐_ *_x_* and Rh_1_/TiO_2‐2×_) in the orange and pink zones, respectively.^[^ ^27^ ^]^ It is found that hydrogen pressure stabilizes both the substitutional and the supported Rh site, but the latter is more stable in the oxygen‐lean conditions. Likewise, the CO adsorption completely changes the thermodynamic site preference for the Rh, driving them to a supported site irrespective of the presence of O vacancies. This is attributed to the much stronger CO adsorption on the supported Rh (−4.89 eV for Rh_1_/TiO_2_) than the substitutional Rh (−2.74 eV for Rh_1_@TiO_2‐2_ *_x_*).

Beyond its position on the support, the stability of SACs versus clustering is another crucial aspect, especially when it is potentially mobile in a reactive atmosphere. Based on Monte Carlo simulations, Myslivecek et al. demonstrated a competition for atoms between Pt single‐atom sites and Pt nanoparticles. This led to dynamic evolution in the Pt nanoparticles population on the ceria surface.^[^ ^12^ ^]^ In an oxidizing atmosphere, Pt single‐atom sites provide strong bonding to single Pt atoms, and Pt nanoparticles shrink. In a reducing atmosphere, Pt single‐atom sites are depopulated, and Pt nanoparticles grow. Very recently, Wang et al. demonstrated the critical roles of CO in the reversible transformations of Pd_1_/CeO_2_ during methane oxidation.^[^ ^6^ ^]^ The presence of CO destabilizes the Pd_1_ single atoms via a Pd_1_‐promoted interfacial reduction, mobilizes the step‐Pd_1_/Pd_ad,_ and stabilizes as‐formed PdO*_x_* subnanometric clusters at the doped terrace sites via a non‐reductive CO adsorption. As shown in Figure 1D–F, cationic single‐atom Pd_1_ in a square‐planar Pd_1_O_4_ conformation is thermodynamically favorable over other states at elevated temperatures in an oxidative atmosphere, providing the tendency of formation and regeneration of Pd_1_ single atoms in the fresh and spent catalysts.^[^ ^6^ ^]^ Upon the exposure of CO, Pd_1_ could facilitate the extraction of surface oxygen in its vicinity, leading to a reduced Pd_1_/CeO_2_ interface even at room temperature. The coordination of Pd_1_ at the terrace site remains Pd_1_O_4_, whereas step site Pd_1_ is reduced to Pd_1_O_2_. This indicates that two (terrace) to three (step) O per Pd atom are extracted from the interface, leaving Pd_1_^δ+^ and Pd_1_O_2_ at steps and partially reduced Pd_1_O_4_ at terraces. Additional CO adsorbed on step Pd_1_^δ+^ serves as a diffusion promotor, substantially increasing the mobility of surface Pd compared with Pd_1_^δ+^ in the absence of CO. Terrace Pd_1_ remains immobile and serves as anchoring sites for CO–Pd_1_ originating from step sites, leading to the formation of Pd_2_/Pd_3_ and nucleation of PdO*_x_* subnanometric clusters. In contrast, the energy barrier for such nucleation is much higher in the absence of CO (2.4 vs 0.1 eV). Finally, the single‐site Pd_1_/CeO_2_ regenerates at high temperatures owing to its thermodynamic stability. Such reversible transformations in the Pd_1_/CeO_2_ catalyst are modulated by the dynamic working conditions (temperature and atmosphere).^[^ ^6^ ^]^

### 2.5 Influence of Catalyst Support

**Figure 2:**
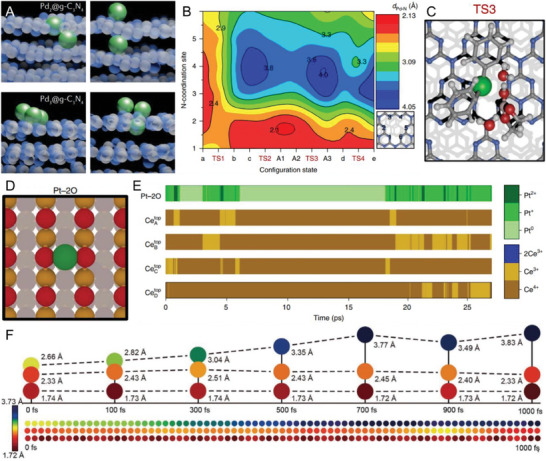
Adaptive coordination environment in SACs. A) Snapshots of molecular dynamics simulations of palladium dimers and trimers stabilized on *g*‐C_3_N_4_, illustrating distinct possible configurations; B) Contour plot of the interatomic Pd–N distances for each N‐coordination site at each of the intermediate and transition states; C) The transmetallation step (A2 → A3) for Pd‐ECN; D) Coordination environment of Pt–2O, where two O atoms are acting as ligands; E) Time evolution at 600 K of the atom‐resolved oxidation states of the Pt and surface Ce atoms for Pt–2O; F) AIMD calculations of the structure of the *CO step of the catalytic process with respect to the change in the NiZn–N_6_–C, Ni–N_4_–C, and Zn–N_4_–C bond lengths with time. Reproduced with permission.^[^ ^1^, ^29^, ^31^, ^40^ ^]^ (A‐E) Copyright 2018, 2021, 2023. Springer Nature. (F) Copyright 2021, Wiley‐VCH.

Practically relevant host materials typically have irregular 3D morphologies and exhibit non‐uniform surface structures and compositions, resulting in high polydispersity of coordination sites for SACs.^[^ ^1^ ^]^ Therefore, several energetically favorable configurations may coexist with minimal interchange barriers. As illustrated by molecular dynamics (MD) simulations in **Figure** **2**A, a wide range of surface and subsurface configurations of palladium atoms, dimers, and trimers were identified on the exfoliated carbon nitride (ECN) scaffold.^[^ ^28^ ^]^ In particular, the flexible lattice of ECN enables an almost continuously variable coordination pattern of palladium to the host during the catalytic cycle of Suzuki coupling.^[^ ^29^ ^]^ As shown in the density functional theory (DFT) calculations in Figure 2B,C, the initial catalyst of Pd_1_‐ECN has a coordination number of 6 and gradually reduces to 3.2 upon the adsorption and exothermic activation of bromobenzene. The Pd atom is less coordinated to the matrix during transmetallation (2.6), elimination, and the C─C bond formation (3.0) and recovered to the initial coordination state by the endothermic removal of the product. Such adaptive coordination to the support enables high stability to deactivation by metal leaching in Suzuki coupling.^[^ ^29^ ^]^

Likewise, reducible oxide supports such as TiO_2_, Fe_2_O_3,_ and CeO_2_ are especially suitable for stabilizing single atoms due to the strong metal–substrate interaction.^[^ ^30^ ^]^ The unique Ce^3+^/Ce^4+^ redox properties, associated with the reversible formation of oxygen vacancies, render CeO_2_ a widely used support. A theoretical analysis of platinum atoms on CeO_2_ identified the possible coexistence of several well‐defined and dynamically interconnected charge and oxidation states, demonstrating the oversimplification of the current static picture of electronic structures.^[^ ^31^ ^]^ As shown in the MD simulation in Figure 2D,E, Pt single atoms can be trapped on CeO_2_(100) in the form of Pt^2+^ with four O ligands owing to the inherent surface oxygen mobility. The most robust Pt^2+^–4O reduces to Pt–3O and Pt–2O with a dynamic number of Ce^3+^ centers under a reductive atmosphere, leading to the co‐existence of Pt^0^, Pt^+^, and Pt^2+^ oxidation states with different lifetimes.^[^ ^31^ ^]^ Similarly, Rousseau et al. presented ab initio molecular dynamics (AIMD) simulations of an unprecedented dynamic single‐atom catalytic mechanism for CO oxidation by ceria‐supported gold clusters. Such a mechanism results from the ability of the gold cation to strongly couple with the redox properties of the ceria in a synergistic manner, thereby lowering the energy of redox reactions. The gold cation can break away from the gold nanoparticle to catalyze CO oxidation adjacent to the metal/oxide interface and reintegrate into the nanoparticle after the reaction.^[^ ^32^ ^]^ Such dynamic response in the local coordination environment has also been reported in the Rh_1_/TiO_2_ for reverse water‐gas shift reaction,^[^ ^27^ ^]^ Pt_1_/TiO_2_ for CO oxidation,^[^ ^33^ ^]^ Cu–N–C and Ru–N–C SACs for hydrogen and oxygen evolution, respectively.^[^ ^34^, ^35^ ^]^ Finally, the nature of metal atoms may also affect the stability of SACs due to the self‐catalytic decomposition of the substrate during the catalytic cycle. For metals with excellent catalytic activity (such as Pt), carbon‐based SACs usually suffer from poor long‐term stability arising from the corrosion of the carbon support, dissolution into the electrolyte, and subsequent redeposition (aggregation) of metal atoms on the surface. Harsh conditions (such as high voltage and acidic electrolyte) can also accelerate such corrosion process, calling for careful consideration of the active metal and more corrosion‐resistant supports.^[^ ^2^ ^]^

### 2.6 Multimetallic and Multinuclear Systems

The stability of bimetallic and multimetallic SACs has received less attention. Besides changes in size, segregation phenomena can occur and be evaluated by DFT using the segregation and aggregation energies as descriptors of heteroatom mobility.^[^ ^1^ ^]^ Such atomic arrangement could occur continuously toward alloying, dealloying, or segregation determined by the element type, atomic ratio, and external conditions in the phase diagram.^[^ ^23^, ^36^ ^]^

With the development of SACs from monometallic to multimetallic systems, the interaction between individual atoms has received increasing attention in regulating electronic structures and catalytic reactivity.^[^ ^37^ ^]^ Such metal–metal interaction becomes more prominent in ultrahigh loading SACs, whose inter‐site distance is sufficiently close to enjoy a synergistic effect.^[^ ^37^, ^38^, ^39^ ^]^ In such an event, the bond length of the heteronuclear pair may experience dynamic changes. As shown in Figure 2F, AIMD simulations were performed to track the bond length change in a bimetallic NiZn–N_6_ system during the CO_2_ reduction reaction (CO2RR). It is found that the length of Ni─Zn, Ni─N, and Zn─N bonds change in a certain range with the formation of COOH^*^ and CO^*^ intermediates, confirming the synergistic effect of Ni─Zn bimetal sites in the kinetic pathway.^[^ ^40^ ^]^ The adaptable coordination of individual Cu sites in the Cu_g_/PCN catalyst also enables a cooperative bridge‐coupling pathway through dynamic Cu─Cu bonding.^[^ ^41^ ^]^ This will be detailed in Section 3.4.

## 3 Influence of Dynamic Aggregation in the Catalytic Cycle

Dynamic aggregation can be beneficial or deleterious on catalytic performance, depending on the optimal initial state. We will highlight the influence of dynamic aggregation on catalytic activity, selectivity, and stability in the following section. Note that such structural dynamism may vary under different catalytic reactions (such as photocatalysis, electrocatalysis, and gas‐phase reactions) due to variations in external driving force (Section 2.2). It should be carefully examined for a comprehensive understanding.

### 3.1 Catalytically Active Site: Clusters or SACs?

**Figure 3:**
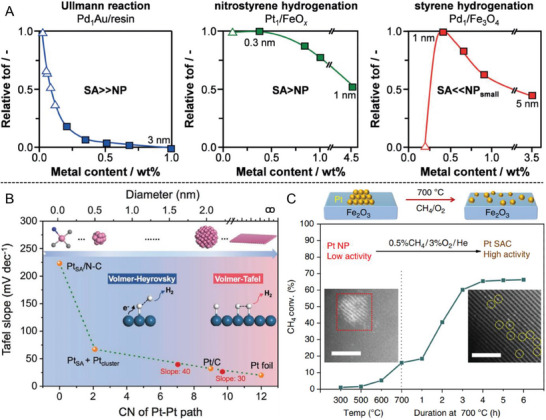
Influence of dynamic aggregation on catalytic activity. A) Trends in relative TOF upon reducing the atomic population of metal nanoparticles and SACs; B) Quantitative correlation between the first‐shell Pt–Pt coordination numbers of various Pt catalysts and corresponding Tafel slope for the HER; C) Dynamic formation of a Pt SAC during methane oxidation. Light‐off curve of 1Pt/Fe_2_O_3_‐NP for methane oxidation. STEM images of catalyst before (left) and after (right) reaction. Scale bars, 2 nm. Reproduced with permission.^[^ ^17^, ^42^, ^43^ ^]^ (A) Copyright 2018, Wiley‐VCH. (B) Copyright 2021, Wiley‐VCH. (C) Copyright 2019, Springer Nature.

The determination of catalytically active sites in subnanometric metal catalysts is challenging owing to their structural dynamism.^[^ ^2^ ^]^ Particularly, they may have completely different activities toward the same reaction. It has been reported that, in some reactions, SACs are the active species, while clusters and nanoparticles are not. In such events, a sharp increase in catalytic turnover will be observed at low metal content when the majority of metal centers become spatially isolated (Ullmann reaction in **Figure** **3**A).^[^ ^9^, ^42^ ^]^ If nanoparticles can also catalyze the reaction, the activity will rise more gradually with increasing dispersion until it reaches a plateau due to the maximized atom utilization (Pt_1_/FeO*_x_* for nitrostyrene hydrogenation). For those reactions where SACs are ineffective (such as Pd_1_/Fe_3_O_4_ for styrene hydrogenation), size reduction will lead to diminished activity as metal clusters are no longer present in the sample.^[^ ^42^ ^]^ Additionally, an induction period may appear when the “real” catalyst is not initially added into the system, while transformation into inactive species results in a decline in activity in a prolonged reaction period.^[^ ^2^ ^]^

To further understand the imperative role of structural dynamism, a correlation between the coordination number of Pt–Pt and the corresponding Tafel slope in hydrogen evolution reaction (HER) was established for a series of Pt‐based catalysts in Figure 3B.^[^ ^43^ ^]^ Atomically dispersed Pt SACs possess a substantially high Tafel slope and undergo restructuring into tiny clusters due to weakened Pt–N bonding under cathodic potentials. This leads to drastically decreased Tafel slopes and a transition from the Volmer‐Heyrovsky pathway to the Volmer‐Tafel pathway when the particle size increases to 2 nm or even larger. Such tiny clusters are responsible for the improved HER activity and stability rather than the initial Pt SACs.^[^ ^43^ ^]^ Likewise, the electrochemically reconstituted Cu_4_ clusters from Cu_1_/CeO_2_ are the real active sites for electrocatalytic urea synthesis due to favorable C–N coupling reactions and urea formation.^[^ ^44^ ^]^ An in situ formed Cu(I)/Cu(0) mixture from the initial Cu_1_/TiO_2_ can effectively promote the generation of CH_4_ in the photocatalytic CO_2_ reduction.^[^ ^45^ ^]^ The breakage of the Rh–Rh bond into the mononuclear complex in supported Rh nanoparticles could be facilitated by the iodine radical and CO molecules in the working condition.^[^ ^46^ ^]^

Conversely, the redispersion of clusters into single atoms could also be beneficial. As shown in Figure 3C, the on‐stream activity of 1Pt/Fe_2_O_3_ nanoparticles displayed a monotonic increase in 4 h at 700 °C to reach a 65% conversion for methane oxidation. This was accompanied by the disappearance of nanoparticles and the formation of atomically dispersed Pt in the spent catalyst in Figure 3C.^[^ ^17^ ^]^ Such phenomenon is more likely to occur in thermally stable SACs for gas‐phase reactions at high temperatures, including the Pd–N_4_ SAC for semihydrogenation of acetylene,^[^ ^16^ ^]^ atomically dispersed CuO*_x_* for deNO*_x_* reactions (NO + CO or NH_3_ + NO + O_2_),^[^ ^7^ ^]^ Ru_1_/MAFO catalysts for N_2_O decomposition,^[^ ^18^ ^]^ and Pd_1_@CeO_2_ core–shell catalysts during calcination in O_2_.^[^ ^47^ ^]^

Apart from clustering, the role of SACs in heterogeneous catalysis is sometimes questioned by the distinct possibility of metal leaching in the presence of a ligand or solvent.^[^ ^3^ ^]^ The homogeneity or heterogeneity of SAC‐promoted reactions should be carefully examined by several critical criteria as previously discussed, which will not be detailed due to limited scope.^[^ ^3^ ^]^

### 3.2 Modulated Selectivity via Dynamic Aggregation

**Figure 4:**
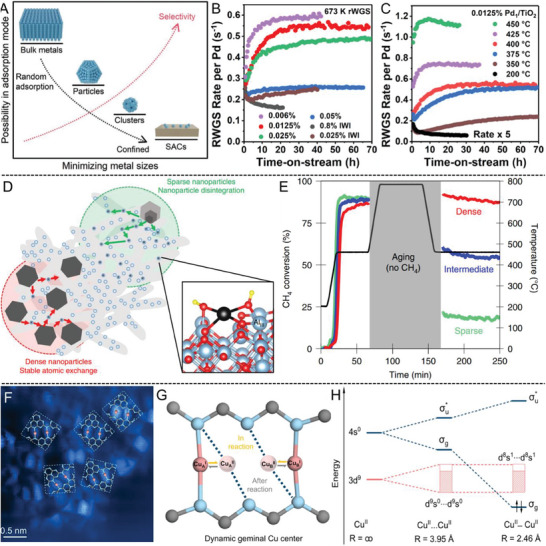
Influence of dynamic aggregation on selectivity and stability. A) The relationships of possible adsorption configuration of reactants and reaction selectivity per metal atom on a traditional supporting substrate with metal size; B) Variations in the RWGS rate per Pd with TOS at 400 °C for Pd/TiO_2_ catalysts at various Pd loadings; C) 0.0125 wt% Pd_1_/TiO_2_ at various temperatures; D) Statistical mechanics model of density‐dependent particle decomposition; E) CH_4_ conversion profiles for Pd/Al_2_O_3_ catalysts with different nanoparticle loadings following the temperature profile; F) STM characterization of Cu_g_/PCN at 370 K; G) Schematic illustration of the dynamic coordination of geminal Cu active centers for C–O coupling over Cu_g_/PCN; H) The energy levels of Cu (II)···Cu (II) interaction with increasing proximity from infinite distance to 2.46 Å; Reproduced with permission.^[^ ^3^, ^41^, ^53^, ^57^, ^58^ ^]^ (A) Copyright 2021, Wiley‐VCH. (B, C) Copyright 2023, ACS. (D, E) Copyright 2019, Springer Nature. (F–H) Copyright 2023, Springer Nature.

SACs are known to be chemoselective owing to the restricted adsorption configuration in contrast to the diverse binding sites on conventional heterogeneous catalysts (**Figure** **4**A). This allows rapid replication of molecular catalysis for the synthesis of complex molecules.^[^ ^3^, ^48^ ^]^ However, as every coin has two sides, this could be problematic when selectivity regulation toward specific products is needed for SACs.^[^ ^2^ ^]^ Beyond conventional strategies via modulating the electronic structure and coordination environment, (reversible) dynamic aggregation offers a distinct advantage in breaking the selectivity limitation, particularly for those reactions that require adjacent metal sites (such as CO2RR^[^ ^49^ ^]^ and complete oxidation of hydrocarbons^[^ ^6^ ^]^).

Take the CO_2_ reduction reaction (CO2RR) as an example; most SAC catalysts tend to convert CO_2_ into C1 products such as HCOOH, CH_3_OH, and CH_4_ but fail to obtain the more profitable C2^+^ hydrocarbon products (ethanol, ethene, etc.) due to the lack of nearby active sites for the C─C coupling steps between two ^*^CO intermediates.^[^ ^37^, ^49^ ^]^ Combining the experimental data and DFT calculations, Xu et al. revealed the reversible transformation of Cu SACs to Cu*_n_* clusters (*n*  =  3 and 4) under CO2RR potentials, thus achieving a CO_2_‐to‐ethanol Faradaic efficiency (FE) of 91% at −0.7 V and an onset potential of −0.4 V.^[^ ^49^ ^]^ This is further validated by Karapinar et al. using operando X‐ray absorption (XAS) under the working conditions of CO2RR.^[^ ^50^ ^]^ For the gas‐phase hydrogenation of CO_2_, the single‐atom Cu–Zr catalyst with Cu_1_‐O_3_ units is the sole contributor to the desirable methanol. Meanwhile, the presence of small copper clusters or nanoparticles leads to the formation of CO by‐product.^[^ ^51^ ^]^ Similarly, such dynamic aggregation of Pt SACs to nanoparticles will induce the switching of the two‐electron pathway to the four‐electron pathway in the oxygen reduction reaction (ORR), producing H_2_O instead of H_2_O_2_. The reason lies in the side‐on adsorption configuration of O_2_ on Pt nanoparticles, in comparison with the end‐on configuration on Pt SACs with a shorter O─O bond length.^[^ ^52^ ^]^ The change in catalytic selectivity via dynamic aggregation is universal to many reactions, whose (irreversible) progressive change with time may lead to catalyst deactivation in Section 3.3.

### 3.3 Catalyst Stability under Operational Condition

Despite numerous efforts, industrial deployment of SACs is still limited due to the pursuit of better activity and selectivity at the expense of stability.^[^ ^2^ ^]^ As mentioned in Section 2.1, the instability of SACs is primarily attributed to the high surface energy of single metal atoms, prompting them to aggregate into more stable nanoparticles (sintering). Other deactivation pathways include the oxidation of carbon support, metal leaching, amorphous carbon deposition, and poisoning in operational conditions.^[^ ^3^ ^]^ A unified framework on catalyst deactivation was established in a comprehensive review by Pérez‐Ramírez et al., detailing the types and approaches used to study deactivation phenomena across all catalyst types and driving forces.^[^ ^8^ ^]^ Herein, we will highlight the influence of dynamic aggregation on the stability of SAC‐mediated processes.

The size effect on the stability profile under working conditions is illustrated in Figure 4B,C using the Pd/TiO_2_‐anatase SAC catalyzed RWGS reaction as an example.^[^ ^53^ ^]^ A progressive, two‐fold increase in the RWGS rate per Pd with time‐on‐stream was observed in the ≤0.025 wt% Pd_1_/TiO_2_ samples (purple, red, and green curves). Such increases originate from higher intrinsic activity of Pd instead of higher Pd dispersion, as Pd is already atomically dispersed on fresh Pd_1_/TiO_2_. In contrast, this was not found in less dispersed Pd/TiO_2_ at higher loadings. More detailed studies revealed the partial sintering of single Pd atoms (Pd_1_) into disordered Pd clusters (Pd*_n_*, ≈ 1 nm) by H_2_ activation and the redispersion into Pd_1_ in an oxidative atmosphere. Such dynamic aggregation is responsible for the increasing rate with time‐on‐stream, and steady‐state Pd active sites are similar to the ones formed under H_2_. Meanwhile, the sintering of Pd_1_ into larger crystalline Pd nanoparticles (≈5 nm) during CO treatment will deactivate the catalyst, leading to progressive activity loss in the time profile.^[^ ^53^ ^]^ This indicates the importance of partial sintering into redispersible clusters for an optimized stability.^[^ ^1^, ^8^ ^]^ Similar observations were reported in the 0.17% Pt@MCM‐22 for NO reduction with CO and H_2_,^[^ ^54^ ^]^ Pd_1_/CeO_2_ for methane oxidation,^[^ ^6^ ^]^ Ni@1T‐MoS_2_ for hydrogen evolution,^[^ ^55^ ^]^ Cu_1_/CeO_2_ for electrocatalytic urea synthesis,^[^ ^44^ ^]^ etc. In an extreme case, unprecedented chemical stability under hydrogenation conditions was shown in the Ni_1_Cu_2_/g‐C_3_N_4_ catalyst without any visible decline in either activity or selectivity for at least 350 h at 160 °C. In sharp contrast, Ni_1_/g‐C_3_N_4_ showed a slight activity increase approximately in the first 6 h and then rapidly deactivated with an activity loss of ≈50% in about 50 h owing to severe agglomeration of Ni atoms into Ni particles.^[^ ^56^ ^]^

Very recently, Cargnello et al. demonstrated an opposite deactivation pathway via the decomposition of nanoparticles into inactive single atoms at high temperatures (Figure 4D), which is remarkably fast and strongly dependent on the particle density and concentration of support defect sites.^[^ ^57^ ^]^ As shown in Figure 4E, the thermal stability of various Pd nanoparticles on Al_2_O_3_ with dense, intermediate, and sparse loadings was probed in methane combustion, where the sparse one was expected to be most stable due to a lower probability of particle migration and coalescence or interparticle atomic exchange. Surprisingly, the dense Pd/Al_2_O_3_ catalyst showed completely stable activity after high‐temperature aging, while the sparse one rapidly deactivated with the conversion decreasing from 85% to 20%.^[^ ^57^ ^]^ Such size dependence strongly suggests that the atomic ripening process is limited by atomic emission related to the existence of a lowest‐energy Pd(OH)_2_ adsorbate atop tri‐coordinated Al atoms in Figure 4D rather than surface diffusion of atomic species to a nearby site. Such nanoparticle‐to‐single atom deactivation process is usually overlooked at low metal loadings.^[^ ^57^ ^]^

Since the influence of dynamic aggregation on the activity, selectivity, and stability is always correlated, a radar plot charting the key catalytic descriptors allows better evaluation of various subnanometric catalysts.^[^ ^58^ ^]^ For alkyne semi‐hydrogenation, the stability could be assessed by the segregation energy (*E*_seg_), which is the differential energy between the ground state (i.e., an alloyed metallic surface) and the system after segregating one atom toward the surface. The activity depends on the adsorption of alkyne and, more importantly, the hydrogen activation energy to split molecular hydrogen, while the selectivity depends on the adsorption energy of the alkene and the ensemble area.^[^ ^58^ ^]^ It is found that the preferential hydrogen adsorption on Pd induces severe segregation effects in Pd_3_@C_3_N_4_ or other Pd nanoparticles. Such Pd segregation or islanding is not favorable in Pd_3_S/C_3_N_4_ due to the polarization of the Pd─S bond, thus outperforming all state‐of‐the‐art catalysts in alkene formation rate and durability with no sign of segregation.^[^ ^58^ ^]^ This phenomenon highlights multiple design criteria that need to be harmonized to develop an effective SAC.

### 3.4 Synergy from Correlated SACs

The switching of the single‐site pathway in SACs to the multi‐site pathway in clusters or particles is a fundamental reason for the modulated performance during structural dynamism. Beyond dynamic clustering, it is also possible for SACs to retain spatial isolation and correlate with the adjacent sites via inter‐site metal–metal interaction.^[^ ^38^, ^39^, ^41^, ^59^ ^]^ Since the number and type of the neighboring atoms in the first and second coordination shells significantly influence the local geometry and charge density of metal centers, such correlated SACs may enjoy unique structural flexibility for dynamic response under working conditions.^[^ ^38^ ^]^

As shown in Figure 4F, Lu et al. recently developed an emerging class of SACs with paired single‐atom sites in specific coordination and spatial proximity (≈4 Å). The Cu_g_/PCN catalyst, called geminal‐atom catalysts (GACs), enables a cooperative bridge‐coupling pathway through dynamic Cu─Cu bonding for diverse C‐X (X  =  C, N, O, S) cross‐couplings with a low activation barrier owing to the adaptable coordination of individual Cu sites.^[^ ^41^ ^]^ Comprehensive theoretical calculations on the plausible mechanisms strongly support the preference of a direct coupling pathway, in which a dynamically formed Cu_2_ dimer with direct Cu─Cu bonding facilitates the C─O bond formation through oxidative addition and subsequent reductive elimination in Figure 4G. The formation of such a dynamic Cu_2_ dimer configuration is energetically compensated through Cu─Cu 4s─4s bonding, accompanied by a potential‐energy‐surface crossing from Cu^II^(d^9^s^0^)…Cu^II^(d^9^s^0^) triplet to Cu^II^(d^8^s^1^)–Cu^II^(d^8^s^1^) singlet states in Figure 4H. These intrinsic advantages of GACs enable the assembly of heterocycles with several coordination sites, sterically congested scaffolds, and pharmaceuticals with highly specific and stable activity.^[^ ^41^ ^]^ Our recent studies also revealed the dynamic process of ultrahigh loading Cu_1_‐C_3_N_4_ SACs in the bimolecular nitrile–azide cycloaddition by operando XAS, triggering the dinuclear pathway when intermetal distance is reduced to the typical diffusion length of the reactants (<1 nm).^[^ ^59^ ^]^ A distance threshold around 5.3 Å between adjacent Ni–N_4_ and Cu–N_4_ moieties is revealed to trigger effective inter‐metal interaction in correlated NiCu SACs for promoted CO2RR activity and selectivity.^[^ ^37^ ^]^ This is consistent with the findings in densely populated Fe SACs for ORR.^[^ ^60^ ^]^

Besides affecting the activity and selectivity, the distance between metal species also determines the collective properties that affect the stability by influencing the propensity towards sintering or redispersion, which will not be further detailed.^[^ ^1^ ^]^

### 3.5 Structural Dynamism in a Confined Space

Subnanometric clusters or single atoms can be restricted in the confined space of zeolite, mesoporous carbon, metal‐organic frameworks (MOFs), and other porous materials using solution synthesis with inorganic salts or organo‐metallic complexes.^[^ ^2^, ^61^ ^]^ The structural dynamism of confined SACs could be significantly different from that of conventional counterparts due to sluggish diffusion kinetics and the limited supply of available atoms in the confined space, as documented by the excellent reviews by Liu and Corma.^[^ ^62^, ^63^ ^]^

For instance, the dynamic structural transformation of subnanometric Pt species confined in MCM‐22 zeolite (including atomically dispersed Pt and Pt clusters) was studied by in situ transmission electron microscopy (TEM) under oxidation‐reduction and reaction conditions.^[^ ^54^ ^]^ Compared with conventional Pt nanoparticles, the behaviors of subnanometric Pt species are much more sensitive to the presence of reactants. Dynamic and reversible transformation between single atoms, clusters, and nanoparticles has been observed under CO and O_2_ reaction conditions at different temperatures. By tuning the size and spatial distribution of Pt species in MCM‐22, subnanometric Pt clusters can be stabilized under reaction conditions, even at very high temperatures (> 800 °C).^[^ ^54^ ^]^ Likewise, the evolution of Cu cations confined in the chabazite zeolite (CHA‐type zeolite) during the selective catalytic reduction (SCR) of NO*_x_* by NH_3_ was studied, showing high mobility of atomically dispersed metal species under reactive conditions. It was proposed that single‐site Cu species can travel through the eight‐membered‐ring window of CHA supercages and form binuclear Cu species as the active sites for low‐temperature NH_3_‐SCR reactions.^[^ ^64^ ^]^ The Pt particles on the external surface of MFI zeolites can disintegrate into subnanometric Pt species and get stabilized in the zeolite channels during high‐temperature calcination in air, while Sn species migrate from surface to internal region during reduction at 650 °C. Dense subnanometric PtSn clusters in the sinusoidal 10MR channels could be formed in the subsequent reduction cycle, enabling highly regioselective propane dehydrogenation.^[^ ^65^ ^]^ This suggests that understanding the confinement effect in structural dynamism is crucial for stabilizing the active sites under harsh reaction conditions and regenerating the deactivated catalyst.^[^ ^63^ ^]^

## 4 Monitoring the Structural Dynamism via Operando Techniques

In pursuit of the molecular level understanding of reaction mechanisms and better designs of SACs, substantial efforts have been focused on determining catalytically active sites in the dynamic catalytic cycle when interacting with substrate molecules.^[^ ^9^ ^]^ Ex situ characterization of the fresh and spent catalysts (e.g., microscopy, spectroscopy) is generally performed to evaluate the structural and electronic changes. Due to dynamic evolution, such structural features given by these ex situ techniques may not be sufficiently reliable to describe the active sites and structure–property relationship.^[^ ^10^ ^]^ This necessitates monitoring structural dynamism via operando techniques in the following section.

### 4.1 Operando X‐Ray Absorption

X‐ray absorption spectroscopy (XAS) is arguably the most powerful technique in detecting the dynamic profile of SACs during the reaction course, even up to 1000 K and 100 bar.^[^ ^4^ ^]^ In a typical XAS experiment, an atom of the measured element absorbs incident X‐ray photons with energies near or above the core level binding energies of that atom. Electrons would then escape from their quantum level and carry with the excess energy as kinetic energy if the energy of X‐ray is larger than electronic binding energy. The corresponding XAS spectrum is acquired by recording the X‐ray energy and adsorption intensity.^[^ ^23^ ^]^ Therefore, the change in the valence state of the active metal is reflected in the X‐ray absorption near edge structure (XANES) region, while extended X‐ray absorption fine structure (EXAFS) provides fruitful information on the local coordination environment in the bulk materials (such as spatial distribution, bonding conditions, and coordination numbers).^[^ ^3^ ^]^ Typically, the absorption of the incident X‐ray beam is monitored by the attenuation of the transmitted photon beam or via fluorescence as the core holes decay in an operando XANES cell (with Kapton windows) for gas‐phase and electrochemical measurements.^[^ ^10^ ^]^ To prevent corrosion of organic solvent and aggressive reactant on the Kapton window, we have also customized a coin‐cell type reactor with double‐side Al coating for operando XAS in liquid‐phase organic transformations.^[^ ^59^ ^]^

**Figure 5:**
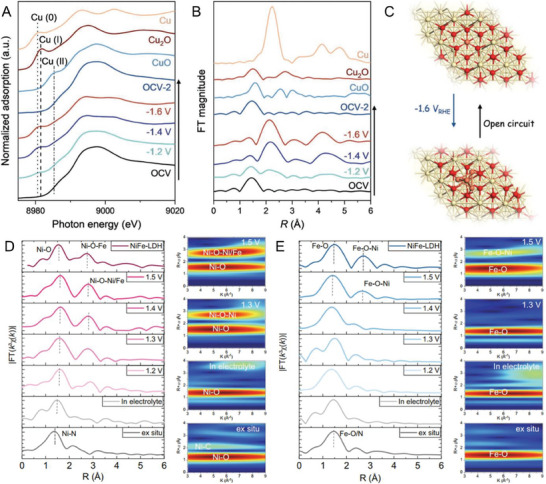
Operando EXAFS for dynamic structural evolution. A) Cu *K*‐edge XANES spectra and B) R‐space EXAFS spectra of Cu_1_–CeO_2_ recorded at different cathodic potentials during C–N coupling; C) Schematic diagram of reconstitution of copper single‐atoms to clusters suggested by the operando XAS measurements; D) FT‐EXAFS and wavelet transform spectra of NiFe‐CNG for Ni and E) Fe K‐edge XAS under OER conditions. Reproduced with permission.^[^ ^44^, ^66^ ^]^ (A‐C) Copyright 2023, Wiley‐VCH. (D, E) Copyright 2021, Springer Nature.

Dynamic aggregation of Cu_1_–CeO_2_ SACs into Cu_4_ clusters during C─N coupling was monitored by operando Cu K‐edge XANES spectra in **Figure** **5**A.^[^ ^44^ ^]^ The formation of Cu(0) and Cu(I) species at different cathodic potentials was validated by the absorption signals located at lower energy edges than those of Cu_1_–CeO_2_ under open circuit voltage. Further analysis by the EXAFS spectra in Figure 5B confirmed the evolution of first‐shell coordination from Cu–O in the Cu_1_–CeO_2_ SAC to mainly Cu─Cu bonding in Cu_4_ clusters. The Cu─Cu bonding was strengthened at more negative voltages (−1.6 V) to a coordination number of 7. Even extending the duration at – 1.6 V to 50 mins, atomically dispersed copper can only aggregate to Cu_4_ clusters, not copper nanoparticles. The reversible transformation of Cu_4_ to Cu_1_ configurations occurred after the removal of voltage, as depicted in Figure 5C. Such electrochemically reconstituted Cu_4_ clusters are genuine active sites for C─N coupling and urea formation, leading to a benchmark urea yield rate of 52.84 mmol h^−1^ g_cat_^−1^ at –1.6 V.^[^ ^44^ ^]^

Likewise, the dynamic profile of dual‐site NiFe SACs in oxygen evolution reaction (OER) was traced by operando XAS in Figure 5D,E.^[^ ^66^ ^]^ It was found that the single Ni sites promoted the key structural reconstruction into bridging Ni‐O‐Fe bonds. Upon increased voltages from 1.2 to 1.5 V, the rising‐edge positions in the Ni K‐edge XANES were positively shifted due to the higher oxidation state of Ni species in NiFe SACs. This was supported by a higher white line intensity. The reversibility with respect to changes in the oxidation state of Ni was confirmed by its partial recovery after the removal of voltage. Meanwhile, the Fe K‐edge XANES only underwent a slight energy shift with increasing applied potentials, probably owing to the electrostatic interaction between catalyst and electrolyte or by OH^−^ adsorption.^[^ ^66^ ^]^ Accordingly, the distance of the first coordination shell of Ni increased at higher applied potentials due to the formation of a greater extent of Ni─O coordination. Another new peak appeared at ≈2.78 Å above 1.3 V, also reflected in the wavelet transformed (WT)‐EXAFS spectra. Similarly, the local coordination of Fe centers steadily evolved at even higher potentials of >1.5 V, indicating that the new bonds are much more difficult to form at the Fe sites than those at the Ni atoms. Such newly formed Ni─O─Fe bonds created spin channels for electron transfer, resulting in a huge improvement in the OER activity with an overpotential of 270 mV at 10 mA cm^−2^.^[^ ^66^ ^]^ A deprotonation process to form the multiple active sites during OER and promote the O─O coupling was observed in atomic iridium stabilized on nanoporous metal phosphides for water oxidation.^[^ ^67^ ^]^ Dynamic evolution of Cu–N_4_ to Cu–N_3_ and further to HO–Cu–N_2_ via the Cu^2+^ to Cu^+^ redox cycle under ORR and CO2RR working conditions was also identified by operando XAS.^[^ ^68^, ^69^ ^]^

The use of operando XAS for dynamic aggregations of SACs is undoubtedly crucial. However, such averaging techniques cannot differentiate local inhomogeneities (polydispersity) arising from a distribution of coordination sites on the support or structural evolution under reaction conditions. This calls for careful interpretation of experimental results.^[^ ^1^ ^]^ The spectroscopic acquisition time is generally several tens of minutes in conventional XAS, which may fail to capture the fast transformation process since most reactions occur rapidly and reach a steady state in a few minutes. This urges the development of time‐resolved XAS techniques to uncover the chemical state evolution of working catalysts.^[^ ^23^, ^70^ ^]^

### 4.2 In Situ Transmission Electron Microscopy

In contrast to averaging spectroscopic methods, microscopies provide spatially resolved information on different catalyst positions in every image frame, with spatial and time resolution depending on the microscope. Transmission electron microscopy (TEM) has long been used to determine particle size distributions (histograms). Recent advances in aberration‐corrected scanning transmission electron microscopy (AC‐STEM), coupled with electron energy loss spectroscopy (EELS), allow the verification of the chemical identity of constituent elements with atomic resolution.^[^ ^10^ ^]^ Visualizing SACs is also essential for understanding the formation, coordination environment, and stability of the catalyst, mainly when the catalytic performance is dominated by dynamic aggregation into other subnanometric species.^[^ ^1^, ^36^ ^]^

**Figure 6:**
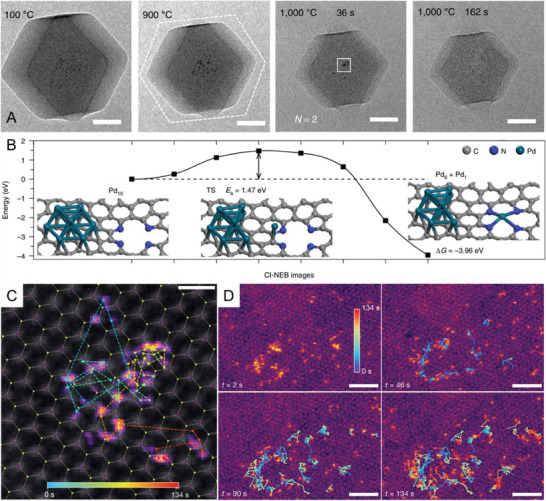
Operando microscopy for dynamic structural evolution. A) Frames acquired at various temperatures and times of Pd‐NPs@ZIF‐8 pyrolyzed in situ with ETEM under an Ar atmosphere. Scale bar: 50 nm; B) Calculated energies along the stretching pathway of the Pd atom from the Pd_10_ cluster to Pd‐N_4_ defect by CI‐NEB, and the corresponding initial and final configurations; C) A single Pt adatom trajectory from a 134 s video in the graphene double liquid cell; D) Trajectories of an ensemble of Pt atoms in the liquid cell, colored according to the elapsed time. Reproduced with permission.^[^ ^16^, ^72^ ^]^ (A, B) Copyright 2018, Springer Nature. (C, D) Copyright 2022, Springer Nature.

The most well‐known example would be the direct observation of transforming nanoparticles into thermally stable SACs via in situ environmental TEM (ETEM), which utilizes a series of apertures and differential pumping to have sections of gradually changing vacuum while protecting the electron gun. Alternatively, it can be achieved with the micro‐electromechanical system (MEMS) based specialty holders to provide stimuli such as heat, gas, light, or strain (or a combination) at the microscale.^[^ ^13^ ^]^ As shown in the representative images acquired at different temperatures and times in **Figure** **6**A, Li et al. provided solid and direct evidence of the evolution from Pd nanoparticles to Pd SACs in a zeolite imidazolate framework‐8 (ZIF‐8) derived nitrogen‐doped carbon substrate via in situ ETEM observations.^[^ ^16^ ^]^ Owing to the coexistence of two competitive atomization and agglomeration processes, initial sintering will occur, leading to a steady increase in the diameter of Pd nanoparticles from room temperature to 900 °C. However, these nanoparticles would eventually transform into single atoms to minimize the *Δ*G of the system. Once the temperature increased to 1000 °C, agglomeration and atomization accelerated. Small Pd nanoparticles vanished in situ at 1000 °C after 36 s (Figure 6A) due to the capture of mobile Pd atoms by N defects on the substrate. Meanwhile, the sintered Pd nanoparticle (6.5 nm in diameter) underwent thermal motions within the substrate and downsized to 4.0 nm at 136 s and 1.9 nm at 150 s by Pd coordination with the N defects before final atomization to single atoms at 162 s.^[^ ^16^ ^]^ This was supported by the highly exothermic (−3.96 eV) process for forming a Pd‐N_4_ SAC from the decomposition of the Pd_10_ cluster in Figure 6B. Such transformation required a moderate energy barrier of 1.47 eV to be overcome, which was much higher than the diffusion energy of a single Pd cluster for sintering (0.58 eV). Hence, sintering was dominant at relatively low temperatures, and atomization dominated at high temperatures (900–1000 °C).^[^ ^16^, ^19^ ^]^

Similarly, the dynamic and reversible transformation between carbon‐supported single Pt atoms and their agglomerates under redox conditions was monitored by in situ TEM.^[^ ^54^ ^]^ In another report, the dynamic process of nanoporous Au to catalyze methane pyrolysis was monitored by in situ TEM, demonstrating the release of Au single atoms by partial disintegration of nanoporous Au surfaces.^[^ ^71^ ^]^ DeRita et al. also systematically investigated the movement of Pt SACs dispersed on anatase TiO_2_ under different reducing and oxidizing conditions via in situ AC‐STEM. The variation in local coordination strongly influenced the chemical reactivity for CO oxidation.^[^ ^33^ ^]^ We should point out that in situ TEM observation under vacuum is only suitable for certain reactions, primarily pyrolysis of SAC precursors and gas‐phase reactions. A complete picture of a catalyst should include operating conditions and investigations into degradation. However, replicating the catalytic environment poses challenges in losing spatial resolution or scattered electrons due to equipment limitations.^[^ ^13^ ^]^ The spatial resolution of in situ TEM is usually around 100 nm, far from the atomic scale. Technical problems such as bubble formation in a liquid environment and electron beam damage need to be solved.^[^ ^23^ ^]^

A typical challenge lies in the TEM observation of SACs in a liquid environment, which is more valuable for applications in (photo)electrochemical and organic catalysis. Conventional liquid cells are made with silicon nitride (SiN*_x_*) windows and are only suitable for observation in nanoparticle formation, dissolution, and shape changes.^[^ ^13^ ^]^ Graphene liquid cells may induce a much thinner liquid layer and less scattering from the windows to achieve atomic resolution in in situ liquid phase imaging. Very recently, Haigh et al. developed a double graphene liquid cell for the monitoring of the dynamics of platinum adatoms on the monolayer in an aqueous salt solution with atomic resolution.^[^ ^72^ ^]^ As shown in Figure 6C,D, a modified adsorption site distribution and higher diffusivities for the adatoms in the liquid phase were found compared with those in vacuum by imaging more than 70 000 single adatom adsorption sites. Most single Pt adatoms are mobile, as demonstrated in the trajectories of several representative Pt adatoms over a sustained period. The high mobility of Pt atoms is consistent with the predominant adatoms located on the surface rather than substituted in the MoS_2_ lattice.^[^ ^72^ ^]^

In summary, microscopy always encounters questions of whether these observations are representative of the whole sample. It should be cross‐referenced by other spectroscopic techniques to exclude the influence of artifacts and beam‐induced damage generated by the experimental conditions. Parameters such as the acceleration voltage, beam current, and pixel dwell time must be optimized to minimize beam‐induced damage and sample drift due to thermal instability or charging. Knock‐on damage and ionization/radiolysis are two major forms of beam damage.^[^ ^13^ ^]^ At higher acceleration voltages, knock‐on damage is more prominent and induces sputtering from the surface, atomic deformation, and defects. Lowering the acceleration voltage below twice the threshold for atomic displacement can effectively reduce such damage. For covalent and weakly ionic compounds at lower acceleration voltages, the principal form of damage is radiolysis due to inelastic scattering, which can readily disrupt the chemical bond and shape.^[^ ^13^ ^]^ Apart from this, microscopic data at atomic resolution tends to be noisy from the low signal‐to‐noise ratio, urging future technological development.^[^ ^13^, ^36^ ^]^

### 4.3 Operando DRIFTS

Diffuse reflectance infrared Fourier‐transform spectroscopy (DRIFTS) using CO or another adsorbate as a probe molecule is a versatile operando method to characterize the electronic structure and nuclearity of the surface metal species in working catalysts.^[^ ^10^, ^30^ ^]^ Specifically, CO has a strong binding to many metal centers, whose site‐specific vibrational fingerprint in 1800– 2200 cm^−1^ depends on the metal oxidation state and local coordination. For instance, the different binding modes (e.g., linear, bridged, and geminal) of CO induce changes in the vibrational frequency, thus providing structural information about the adsorption site.^[^ ^13^ ^]^

**Figure 7:**
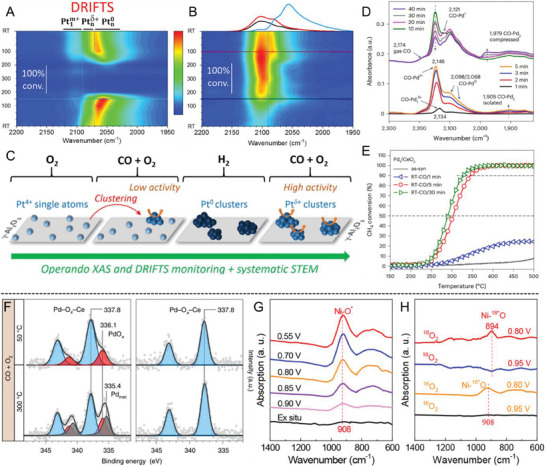
Operando spectroscopies for dynamic structural evolution. A) DRIFTS color maps showing the evolution of the υ_C−O_ absorption band(s) in the Pt carbonyl wavenumber region during postreduction and B) postcalcination reaction steps; C) Schematic illustration of the dynamic structural evolution process; D) In situ DRIFTS during RT‐CO activation; E) CH_4_ oxidation on Pd_1_/CeO_2_ after RT‐CO activation for 1–30 min; F) In situ NAP‐XPS of the Pd 3d core line as a function of the reaction conditions for 1PdRods (left) and 1PdFSP (right); G) Operando SR‐FTIR measurements in the range of 600−1400 cm^−1^ under various potentials for Ni_1_−NC during the ORR process and H) the corresponding isotope‐labeling experiment. Reproduced with permission.^[^ ^6^, ^30^, ^73^, ^77^ ^]^ (A–C) Copyright 2019, ACS. (D, E) Copyright 2023, Springer Nature. (F) Copyright 2021, Springer Nature. (G, H) Copyright 2020, ACS.

As shown in **Figure** **7**A–C, the dispersion, oxidation state, and CO oxidation activity of Pt/*γ*‐Al_2_O_3_ SAC were simultaneously monitored by operando XAS and DRIFTS, which confirmed the dynamically aggregated Pt clusters from single atoms as the active sites for CO oxidation.^[^ ^73^ ^]^ The main peak of CO‐DRIFTS at 2100–2106 cm^−1^ is ascribed to the linearly adsorbed CO on single atoms, while the shoulder at 2070–2090 cm^−1^ present in heating and cooling profile can be ascribed to adsorption on partially oxidized Pt clusters. Single Pt atoms can hardly accommodate CO and oxygen, and the adsorption competition is favorable to oxygen at low temperatures and CO at higher temperatures, which may prevent the SACs from catalyzing the reaction. As the clusters are more active than the single atoms, their formation during the reaction increases CO oxidation activity.^[^ ^73^ ^]^ Likewise, CO‐DRIFTS was conducted to probe the evolution of surface Pd species in methane oxidation (Figure 7D,E).^[^ ^6^ ^]^ After initial saturation of CO on isolated Pd_1_ *^δ+^* and Pd^2+^ at 2134 and 2145 cm^−1^ in the first 5 min, peaks from linear CO adsorption on Pd^0^ gradually appear at 2098 and 2068 cm^−1^, indicating the nucleation of highly dispersed PdO*_x_* clusters containing mixed Pd^2+^/Pd^0^ oxidation states. The presence of highly dispersed Pd species such as Pd_2_ or Pd_3_ is suggested by another weak peak at 1905 cm^−1^ from the CO adsorption on either bridge sites (CO‐Pd_2_) in an isolated state or three‐fold sites (CO‐Pd_3_) on an extended Pd surface. The peak from CO adsorbed on Pd^2+^ slowly decreases after 10 mins, accompanied by the formation of a new peak at ≈1980 cm^−1^ from CO adsorption on Pd_2_ in a compressed state. This suggests the progressive change of Pd_1_ single atoms in larger PdO*_x_* clusters.^[^ ^6^ ^]^ Similar phenomena were observed in the CO‐DRIFTS for quantifying the relative populations of Pd_1_, Pd_n,_ and Pd_p_ within different catalysts. The singular 2040 and 2060 cm^−1^ peaks are assignable to linearly bonded CO on Pd clusters and particles, while the broad bands at lower‐frequency regions (<2000 cm^−1^) are related to the bridge‐bonded or triple‐bonded CO on continuous palladium ensembles. The coexistence of Pd1 in these samples is confirmed by the linearly bonded CO at 2080 and 2120 cm^−1^.^[^ ^30^, ^74^ ^]^

Apart from the CO probe, the adsorption configuration of other reactant(s) on SAC is also somewhat different than that on surfaces of metal nanoparticles. For instance, our Pt_1_‐MoS_2_ prefers a monodentate “end‐on” adsorption of the nitro functionality in 3‐nitrostyrene in the in situ DRIFTS measurements, compared to the planar binding configuration of the alkene groups (such as ethylidyne (tridentate) or di‐σ (bidentate) configuration) on the Pt nanoparticles. This leads to an extraordinary chemoselectivity of 99% toward 3‐aminostyrene.^[^ ^75^ ^]^ In situ DRIFTS of C_2_H_2_ hydrogenation on the spent Ni_1_Cu_2_/*g*‐C_3_N_4_ catalyst confirms an identical result with the fresh sample, validating the excellent stability.^[^ ^56^ ^]^

### 4.4 Near‐Ambient Pressure X‐Ray Photoelectron Spectroscopy

Near‐ambient pressure X‐ray photoelectron spectroscopy (NAP‐XPS) is an emerging operando method due to its inherent surface sensitivity resulting from the small inelastic mean path of the photoelectrons. It can be applied to monitor structural evolution in adsorption, segregation, alloying, and coking during ongoing reactions in the few mbar pressure range.^[^ ^10^ ^]^ For example, PdO (or PdO*_x_*) was believed to be the active phase for methane combustion on Pd/Al_2_O_3_ from the ex situ studies. However, operando NAP‐XPS suggests a new surface phase of PdO seeds on a Pd_5_O_4_ surface oxide, governed by a delicate balance of seed formation and methane reduction. Such PdO decomposes, and the reaction proceeds on metallic Pd at 350 °C and above, but with its near‐surface region saturated by dissolved oxygen.^[^ ^6^, ^10^ ^]^ Since the photoelectrons can only travel a few millimeters at mbar pressure, the nozzle of the electron energy pre‐lens has to be very close to the sample (e.g., a pressed catalyst pellet mounted on a sapphire holder) in a typical NAP‐XAS setup. The gas pressure is then reduced by 10^9^ using strong differential pumping until the electrons reach the hemispherical electron energy analyzer.^[^ ^10^ ^]^

As shown in Figure 7F, the surface electronic structure of the working catalysts was followed by in situ NAP‐XPS. Conventional Pd_1_/CeO_2_ catalyst contains two oxidation states during CO oxidation at 50 °C in the Pd 3d core‐level spectrum. The more intense component at 337.8 eV corresponds to the atomically dispersed Pd^2+^ ions covalently bonded to CeO_2_. In comparison, the second one, located at ≈336 eV, is attributed to small PdO*_x_* clusters, indicating the susceptibility of Pd SACs to reduction and agglomeration even under mild reaction conditions. Almost half of the Pd converts into metallic (335.4 eV) and semi‐oxidized (≈ 336 eV) states at 300 °C. On the contrary, the corresponding NAP‐XPS spectra for high‐surface‐area Pd_1_/CeO_2_ SACs using flame spray pyrolysis only contain the Pd^2+^ oxidation state, independent of the CO oxidation reaction conditions.^[^ ^30^ ^]^ The agglomeration of Ir atoms on Fe_3_O_4_ at 960 K during CO oxidation and water‐gas shift reaction was validated by in situ XPS.^[^ ^76^ ^]^ The dynamic evolution of CuO during the reduction/oxidation treatments in deNO*_x_* reactions was also traced by in situ XPS. Complete reduction of CuO to metallic Cu occurred in H_2_ reduction, leading to the shift of the typical Cu^2+^ peak at 934.0 eV to a lower binding energy. The recovery in the XPS profile to its initial state after re‐oxidation in wet air confirmed good reversibility.^[^ ^7^ ^]^ The excellent stability of Ni_1_Cu_2_/g‐C_3_N_4_ catalyst in hydrogenation could be verified by in situ XPS, where the Cu 2p_3/2_ binding energy was invariant at 933.2 eV for the +1 oxidation state, irrespective of the treatments.^[^ ^56^ ^]^ Nevertheless, most NAP‐XPS studies are conducted in the quasi‐operando conditions in gas‐phase, calling for the development of adapting the XPS technique into or close to practical reaction conditions, particularly for electrochemical or liquid‐phase reactions.^[^ ^23^ ^]^

### 4.5 Operando FTIR and Raman

Apart from DRIFTS, operando FTIR and Raman in the liquid phase are valuable vibrational spectroscopy techniques for revealing the active species, phases, and mechanisms. From a fundamental aspect, infrared spectroscopy reflects the change of dipole moment of molecules, while Raman corresponds to the variation in polarizability. Hence, Raman is more frequently employed than FTIR in the structural profiling of inorganic electrocatalysts due to the limitation of infrared energy.^[^ ^10^, ^23^ ^]^ Depending on the transmissivity of the samples, various operando cells, including transmission, diffuse reflectance, and attenuated total reflection (ATR), are commercially available using KBr or CaF_2_ windows and ZnSe or Ge ATR crystals. For transmission and diffuse reflectance, catalyst powders are usually pressed into pellets or small crucibles, while the crystals are coated with thin catalyst films for ATR.^[^ ^10^ ^]^

For instance, operando synchrotron Fourier transform infrared spectroscopies (SR‐FTIR) were performed to uncover the atomic‐level dynamics of active site evolution at the solid‐liquid electrochemical interfaces associated with the reactive intermediates proceeding over a Ni_1_‐NC SAC during ORR.^[^ ^77^ ^]^ As shown in Figure 7G,H, a new absorption band from the surface ^*^O intermediate over the Ni_1_–N_2_ active site at 908 cm^−1^ appears at 0.85 V. Isotope labeling was then performed to clarify the origin of such intermediate. The isotope exchange of ^16^O/^18^O in singlet oxygen (^*^O) leads to a redshift of the vibrational band from 908 to 894 cm^−1^ when ^16^O_2_ gas is replaced by ^18^O_2_, confirming the potential‐driven chemical coupling between the ^*^O_2_ precursor adsorbed on Ni_1_–N_2_ site and an assisted water molecule adsorbed on adjacent N and C sites. This leads to the formation of crucial ^*^O intermediates and the accumulation of O–H species on the catalyst surface, bypassing the conventional, rate‐determining step of O–O dissociation in ORR.^[^ ^77^ ^]^

Structural fingerprints and the metal–substrate interaction in carbon or oxide‐supported SACs could be derived from operando Raman.^[^ ^4^ ^]^ The strong interaction between isolated Pd_1_ and CeO_2_ support is verified by an apparent redshift from 465 to 453 cm^−1^ in the F_2g_ peak of Pd_1_/CeO_2_ in the Raman spectra, originating from the defect‐sensitive, symmetrical stretching vibration of O^2−^ around Ce^4+^. Notably, the F_2g_ peak suddenly blueshifts to 461 cm^−1^ upon RT‐CO treatment in 5 mins, which is attributed to rapid oxygen extraction and nucleation of PdO*_x_* clusters at the Pd/CeO_2_ interface.^[^ ^6^ ^]^ In separate work, a broad peak attributed to an I‐H vibration band at 1460 cm^−1^ is seen in the in situ Raman for single‐atom iodine stabilized on nickel hydroxide when the potential was more negative than the thermodynamic potential of HER.^[^ ^78^ ^]^

Finally, combining two (or more) operando methods is particularly powerful for an in‐depth understanding of dynamic structural evolution, such as combined XAS/DRIFTS/NAP‐XPS,^[^ ^6^, ^7^, ^30^, ^56^ ^]^ XAS/TEM,^[^ ^33^ ^]^ etc. Theoretical calculations based on DFT, MD, or finite element analysis also provide vital structural features on dynamic processes, which may not be accessible via operando techniques.^[^ ^29^, ^31^, ^32^, ^53^, ^79^, ^80^, ^81^ ^]^ A good example lies in the geminal‐atom catalysis for the well‐designed combination of experimental and theoretical approaches.^[^ ^41^ ^]^ Note that probes such as high energy electrons and X‐rays can damage catalysts; conducting low‐dosage and control experiments is essential to minimize the interference (artifacts). New techniques such as quick‐XAS can effectively reduce the exposure time from around 20 minutes in conventional XAS to several seconds, thus reducing the beam damage.^[^ ^70^ ^]^

## 5 Perspective and Conclusion

### 5.1 Regulation of the Dynamic Aggregation

Given the complexity of dynamic profile in a catalytic cycle, precise fabrication of active sites with controlled atomicity and composition is ever‐more fascinating but incredibly complex to achieve. Such catalyst discovery often relies on empirical screening guided by the influence of variations of the synthetic parameters on performance, which is time‐consuming and unable to predict the catalyst response to a reactive environment.^[^ ^1^ ^]^ This calls for reliable methods to regulate the dynamic transformation between subnanometric species. The understanding of cluster chemistry, including their structure, formation pathway, and reactivity, has also made significant processes.^[^ ^82^, ^83^ ^]^ The transformation chemistry between different cluster species has been extensively studied, especially by mass spectrometry as well as other techniques.^[^ ^84^ ^]^ The cluster chemistry knowledge may potentially benefit the regulation of dynamic aggregation.

Recalling the driving force in Section 2.1, the island growth mode (Volmer‐Weber growth) is the primary mechanism for nanoparticle formation due to more favorable enthalpic interaction within the metal itself than with the substrate.^[^ ^85^ ^]^ A trivial solution lies in the thermodynamic regulation of the metal–substrate interaction to prevent nanoparticle formation (i.e., stable SACs). This often leaves too few metal sites available for reactant binding and catalysis, and sintering may still occur when exposed to sufficiently harsh conditions;^[^ ^86^ ^]^ A nontrivial solution is to tune the supply or diffusion kinetics of free atoms via confinement or defect‐engineering, which allows some degrees of freedom for dynamic response and prevents irreversible sintering into large particles.^[^ ^12^, ^25^, ^87^ ^]^

**Figure 8:**
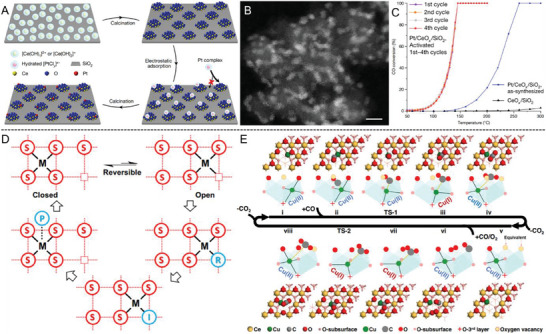
Emerging strategies for controlling the dynamic aggregation. A) Fabrication processes of functional CeO*_x_* nanoglue islands and CeO*_x_*/SiO_2_‐supported Pt_1_ single‐atom catalysts; B) Atomic‐resolution HAADF‐STEM image of crystalline CeO*_x_* clusters; C) Evaluation of low‐temperature CO oxidation activity and stability; D) Possible mechanism with hemilabile coordination in Cu_1_/CeO_2_, where the metal center can migrate from a full metal–support coordination site (the closed state) to a defect site (the open state); E) Optimized structures for Cu_1_/CeO_2_(111) in the catalytic cycle with the hemilabile metal–support coordination. Reproduced with permission.^[^ ^87^, ^89^ ^]^ (A‐E) Copyright 2023, Springer Nature.

To this end, Liu et al. proposed a “nanoglue” strategy via confining atomically dispersed metal atoms on tiny oxide clusters.^[^ ^87^ ^]^ As shown in **Figure** **8**A,B, isolated and defective CeO*_x_* nanoglue islands with a size of less than 2 nm were grafted on high‐surface‐area SiO_2_ as the host for one Pt atom. In contrast to conventional sintering, such Pt SACs remain highly stable under oxidizing and reducing environments at high temperatures and exhibit markedly increased activity for CO oxidation in Figure 8C. This is attributed to the mobility of the Pt atom on each CeO_2_ nanoglue island while preventing inter‐island movement for coalescence. Such a strategy can produce various robust SACs and cluster catalysts.^[^ ^87^ ^]^ Similarly, the confined space in non‐collapse metal‐organic frameworks prevents the excessive aggregation of Cu single atom under cathodic potentials, yielding ultrasmall clusters (≈4 nm) for a benchmark performance in ammonia electrosynthesis via nitrate reduction.^[^ ^88^ ^]^

Mimicking the homogeneous catalyst through a reversible opening and closing of the metal‐ligand coordination sphere in the catalytic cycle is another emerging approach to achieving controllable dynamism. As shown in Figure 8D, Xu et al. proposed a possible mechanism in a heterogeneous Cu_1_/CeO_2_ SAC for CO oxidation, where the metal center can migrate from a fully coordinated site (the closed state) to a defective site (the open state) on the support. The activation of the reactant is facilitated in the open state in the first half of the catalytic cycle (i–iv), while the release of the product is facilitated when the active center is transformed back to the closed state in steps v to viii in Figure 8E. The changes in metal–support coordination are accompanied by the changes in the valence of Cu, i.e., open for Cu(I) and closed for Cu(II).^[^ ^89^ ^]^ Reaction product‐driven restructuring and assisted stabilization at the atomic scale during steam reforming of methane was also observed in the Rh‐on‐ceria catalyst.^[^ ^90^ ^]^ The introduction of such reversible coordination in heterogenous catalysis provides a new perspective for tuning the structural dynamism of SACs.

## 6 Conclusion

Dynamic aggregation into other subnanometric species is a universal behavior in single atom catalysts (SACs) to respond to reactive environments, regardless of their thermodynamic or kinetic stability on support materials. Fundamentally, this is driven by many factors, including changes in the energetics due to interactions with reactants or intermediates and structural transformations of the support. Such aggregation could be progressive and irreversible, leading to a perpetual change in catalytic performance as suggested by conventional wisdom (catalyst deactivation by sintering). By contrast, it may occur discreetly and transiently under reaction conditions, generating redispersible tiny clusters and nanoparticles as the real active sites for various reactions. The restructuring of SACs could be uncovered by combining operando studies using X‐ray absorption spectroscopy, in situ transmission electron microscopy, near‐ambient pressure X‐ray photoelectron spectroscopy, and operando vibrational spectroscopies. In many cases, this is more reliable than those ex situ techniques to describe the active sites and structure‐property relationship due to the complexity of the dynamic profile. It is also crucial to regulate the dynamic transformation between subnanometric species via emerging strategies such as confinement and defect engineering. Such knowledge provides a new paradigm for designing intelligent SACs for practical applications.

## Conflict of Interest

The authors declare no conflict of interest.

## Author Contributions

All authors drafted, discussed, and commented on the manuscript.
